# Management of intrahepatic cholangiocarcinoma: a review for clinicians

**DOI:** 10.1093/gastro/goaf005

**Published:** 2025-01-26

**Authors:** Matteo Colangelo, Marcello Di Martino, Michela Anna Polidoro, Laura Forti, Nastassja Tober, Alessandra Gennari, Nico Pagano, Matteo Donadon

**Affiliations:** Department of Health Sciences, University of Piemonte Orientale, Novara, Italy; Division of Surgery, University Maggiore Hospital della Carità, Novara, Italy; Department of Health Sciences, University of Piemonte Orientale, Novara, Italy; Division of Surgery, University Maggiore Hospital della Carità, Novara, Italy; Hepatobiliary Immunopathology Laboratory, IRCCS Humanitas Research Hospital, Rozzano, Milan, Italy; Division of Oncology, University Maggiore Hospital della Carità, Novara, Italy; Division of Oncology, University Maggiore Hospital della Carità, Novara, Italy; Division of Oncology, University Maggiore Hospital della Carità, Novara, Italy; Department of Translational Medicine, University of Piemonte Orientale, Novara, Italy; Division of Gastroenterology, University Maggiore Hospital della Carità, Novara, Italy; Department of Health Sciences, University of Piemonte Orientale, Novara, Italy; Division of Surgery, University Maggiore Hospital della Carità, Novara, Italy

**Keywords:** intrahepatic cholangiocarcinoma, liver resection, hepatectomy, liver transplantation, chemotherapy, target therapy

## Abstract

Intrahepatic cholangiocarcinoma (iCCA) is an aggressive liver malignancy that arises from second-order biliary epithelial cells. Its incidence is gradually increasing worldwide. Well-known risk factors have been described, although in many cases, they are not identifiable. Treatment options are continuously expanding, but the prognosis of iCCA remains dismal. R0 liver resection remains the only curative treatment, but only a limited number of patients can benefit from it. Frequently, major hepatectomies are needed to completely remove the tumour. This could contraindicate surgery or increase postoperative morbidity in patients with chronic liver disease and small remnant liver volume. In cases of anticipated inadequate future liver remnant, regenerative techniques may be used to expand resectability. The role and extent of lymphadenectomy in iCCA are still matters of debate. Improvements in iCCA diagnosis and better understanding of genetic profiles might lead to optimized surgical approaches and drug therapies. The role of neoadjuvant and adjuvant therapies is broadening, gaining more and more acceptance in clinical practice. Combining surgery with locoregional therapies and novel drugs, such as checkpoint-inhibitors and molecular-targeted molecules, might improve treatment options and survival rates. Liver transplantation, after very poor initial results, is now receiving attention for the treatment of patients with unresectable very early iCCA (i.e. <2 cm) in cirrhotic livers, showing survival outcomes comparable to those of hepatocellular carcinoma. Ongoing prospective protocols are testing the efficacy of liver transplantation for patients with unresectable, advanced tumours confined to the liver, with sustained response to neoadjuvant treatment. In such a continuously changing landscape, the aim of our work is to review the state-of-the-art in the surgical and medical treatment of iCCA.

## Introduction

Intrahepatic cholangiocarcinoma (iCCA) is an epithelial cell malignancy arising anywhere along the biliary tree. It predominantly derives from epithelial cells of the bile ducts, the cholangiocytes; however, it can also develop from peribiliary glands [1–3]. The true incidence of iCCA and other types of cholangiocarcinoma (CCA) is unclear due to prolonged misclassification in national databases [4]. However, iCCA-specific mortality and incidence are increasing worldwide. R0-liver resection remains the only potentially curative treatment. Unfortunately, most patients with iCCA are initially ineligible for surgical resection because of advanced disease at the time of presentation, with only 15% of patients eligible for surgical resection. The widening array of diagnostic and therapeutic tools available and a better understanding of genetic profiles might lead to improved clinical outcomes when patients are evaluated with a multidisciplinary approach [5]. The aim of this review is to summarize the diagnostic work-up and novel treatment options for iCCA.

## Epidemiology

The incidence of iCCA is reported to be higher in Asian countries than in Western ones, especially in Southeast Asia. The highest incidence is reported in Northeast Thailand, with peaks in men of 85 cases per 100,000 [6]. This is due to the fluke-related cancers that increase the incidence of iCCA [7]. However, while iCCA rates in Asia have remained stable over the last few decades, the incidence of iCCA has been steadily increasing in most Western countries [4, 8]. These trends might be explained by revisions of previous versions of International Classification of Diseases (ICD) for iCCA [9], improved diagnostic tools, changing migration patterns in the West [10], and the increasing burden of chronic liver disease which affects Western countries [11, 12].

Well-known risk factors for iCCA are cirrhosis, hepatitis B/C, alcohol consumption, diabetes, obesity, smoking, non-alcoholic steatohepatitis, and liver fluke infestation, as well as biliary diseases, such as primary sclerosing cholangitis (PSC), hepatolithiasis, congenital cholangiectasis (choledochal cyst), Caroli disease, and inflammatory bowel disease. Liver flukes represent a major risk factor in Southeast Asia. The parasites Clonorchis sinensis, Opisthorchis viverrini, and Opisthorchis felineus are infection-associated risks of iCCA [13]. Hence, the epidemiology of iCCA can be investigated as fluke-related and non-fluke-related iCCA in these regions. Infection follows the consumption of undercooked freshwater cyprinid fish carrying the larval parasite. After ingestion, juvenile flukes migrate through the ampulla of Vater into the common bile duct and into the intrahepatic bile ducts. Here they mature, reproduce, and can live for many years [14]. Chronic liver fluke infection is connected with a wide range of hepatobiliary diseases, including inflammation of the gallbladder and bile ducts (cholecystitis and cholangitis), periductal fibrosis, and ultimately, iCCA [3]. The attachment of liver flukes to the biliary wall results in ulceration and the formation of precancerous lesions [14].

The main chemical risk factors are thorium-232, 1,2-dichloropropane, and dichloromethane. A high incidence of iCCA has been observed among employees of a printing company with long-term exposure to these chemical substances [15].

Unfortunately, different studies indicate that no risk factors are identifiable in ∼60%–70% of iCCA cases [16]; as a consequence, prevention and/or surveillance strategies can be only applied to a small subgroup of patients.

## Classification and molecular profiling

**Figure 1.**
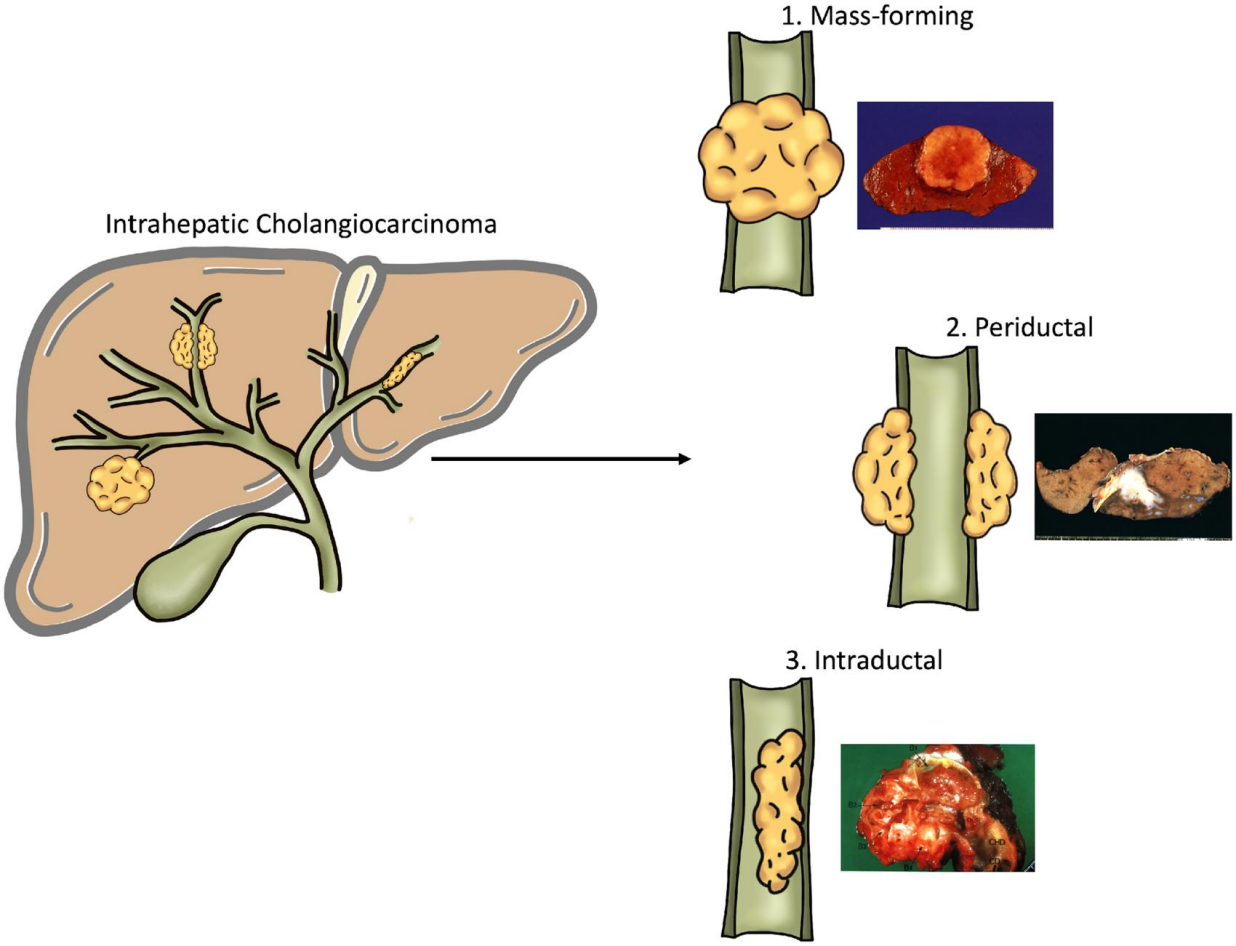
Classification of intrahepatic cholangiocarcinoma (iCCA). Macroscopically, iCCA is classified into three subtypes, (1) mass-forming: iCCA with nodular/mass aspect; (2) periductal infiltrating: iCCA infiltrating along the bile duct; (3) intraductal growing: iCCA growing inside a bile duct. For each type, the corresponding pictures of the surgical specimens are detailed.

According to the 11th version of the International Classification of Diseases (ICD-11), published in 2018, CCA is divided into iCCA, perihilar CCA (pCCA), and distal CCA (dCCA) (Figure 1). Macroscopically, iCCA is classified into four subtypes: (i) mass-forming (MF): iCCA with nodular/mass aspect; (ii) periductal infiltrating (PI): iCCA infiltrating along the bile duct; (iii) intraductal growing: iCCA growing inside a bile duct; (iv) mixed form: iCCA infiltrating along the bile duct with concomitant invasion into close liver parenchyma [17, 18].

Looking at the histological classification, according to the 5th World Health Organization classification, iCCA is split into two different subtypes: the large duct type and the small duct type, with specific clinicopathological features and mutation profiles [17]. Subtyping of iCCA is useful nowadays for predicting clinical outcomes and treatment choice. The large duct type shows a clear glandular structure, with mucin production combined with desmoplastic reaction. In contrast, the small duct type never shows mucin production. Clinically, the large duct type is typical of iCCA PSC-related or originates within the context of hepatolithiasis and liver fluke infection. On the contrary, the small duct type often arises in non-biliary chronic liver disease, like viral hepatitis and metabolic syndrome [17]. Different genetic alterations amenable to targeted therapies characterize the two subgroups [18]: the small duct type is known to harbour isocitrate dehydrogenase (IDH-1 and IDH-2) mutations and fibroblast growth factor receptor (FGFR) fusions. On the other hand, the large duct type often shows KRAS and SMAD4 mutations, typical of pCCA and dCCA subtypes too. Long-term outcomes and survival are better in the small duct type compared to the large duct type [19, 20], which generally shows more aggressive pathological features, such as lymphatic and/or perineural invasion.

## Diagnostic workup

### Clinical presentation

Most patients with iCCA do not have specific symptoms related to the presence of cancer, and liver masses are frequently discovered incidentally during clinical evaluation for other reasons. Early-stage cholangiocarcinoma rarely shows clear symptoms. However, as the disease advances, a variety of symptoms may appear. Weight loss and malaise are often signs of more advanced disease [21, 22]. Some of the most typical signs of cholangiocarcinoma include jaundice, abdominal pain, nausea/vomiting, and fever. Early jaundice can occur if the tumour is located centrally within the liver, obstructing the biliary confluence by extrinsic compression or direct invasion.

### Laboratory test

Liver function should be assessed with measurement of platelet count, serum levels of bilirubin, gamma-glutamyl transferase, aspartate transaminase, alkaline phosphatase, albumin, total protein, and prothrombin time or international normalized ratio. Any abnormality in hepatic function, particularly signs of underlying chronic liver disease, should be investigated in more detail.

Carbohydrate antigen 19-9 (CA 19.9) and carcinoembryonic antigen are the most widely used serum markers used in the diagnosis and monitoring of iCCA. Regarding the role of CA 19.9, a meta-analysis confirmed a sensitivity of 72% and specificity of 84% in distinguishing patients with cholangiocarcinoma and benign biliary disease [23, 24]. Markedly elevated levels of CA 19.9 are associated with poorer prognosis and this marker can also be useful for assessing response to treatment [25]. Interestingly, ∼10% of the general population is unable to produce CA 19.9: in such patients, the marker cannot be used as a diagnostic or follow-up tool [26].

### Imaging

iCCA has a non-specific appearance with the MF type on ultrasound, usually showing as a hypoechoic hepatic mass, whereas the PI type presents as a small mass-like lesion with diffuse periductal thickening and distal dilation [27, 28]. Satellite lesions may be seen on USA, as well as capsular retraction [29]. USA also has potential to identify biliary dilatation, portal venous, and hepatic venous invasion, or, rarely, portal lymphadenopathy [30]. Contrast-enhanced ultrasound (CEUS) can add some additional information, showing heterogeneous enhancement of the tumour margin in the arterial-dominant phase and a marked defect in the late phase. The diagnostic performance of CEUS for iCCA is 60%–90% sensitivity and 65%–98% specificity [31, 32]. CEUS may be beneficial for differential diagnosis between iCCA and hepatocellular carcinoma, thanks to the presence of peripheral arterial enhancement commonly observed in iCCA. However, for tumours less than 3 cm, CEUS could lose part of its effectiveness due to complete enhancement of the lesion [33].

Contrast-enhanced computed tomography (CT) and magnetic resonance imaging (MRI) are the most used diagnostic tools to identify iCCA; however, there are no accepted criteria for definitive non-invasive diagnosis based on imaging alone.

On non-contrast CT, iCCA is usually hypointense and shows a ring-like enhancement pattern in the early phase of contrast-enhanced CT, with a sensitivity and specificity of 60% and 65.5%, respectively [34, 35]. A characteristic persistent enhancement pattern in the late phase (3–6 min following contrast injection) is observed in 67% of cases and depends on the rich stromal component in the middle of the tumour [36]. CT is helpful in identifying portal and hepatic venous involvement [35] and lobar atrophy associated with longstanding biliary or portal venous involvement. CT also has the power to determine the extent of locoregional disease and metastatic spread, which are fundamental features to assess resectability. Data from newer multiphase, fast acquisition scanners can also be used to create 3D models of hepatic and tumour anatomy to improve preoperative surgical planning, as well as perform an accurate assessment of hepatic volumetry in order to decrease the risk of postoperative liver failure [37].

On MRI, the lesion is hypointense on T1-weighted images and mild to moderately hyperintense on T2-weighted images, because of the typical content of desmoplastic stroma and mucin. Findings of dynamic MRI using extracellular fluid contrast agents are similar to those of CT [38] with pooling of contrast within the lesion on delayed images, 6–8 min after contrast injection [36]. PI tumours demonstrate diffuse periductal thickening with enhancement along with biliary structuring and proximal biliary dilation [28]. On diffusion-weighted imaging, the lesion shows concentric hyperintensity in 29%–52% of cases [39, 40]. Cholangiopancreatography sequences are essential in defining biliary anatomy to facilitate resection and reconstruction, if needed. Differential diagnosis with benign intrahepatic fibrous strictures is challenging even for expert radiologists. Similarly, the differentiation between HCC and iCCA in cirrhotic patients is often demanding, owing to their similar findings. The sensitivity and specificity of MRI for differentiating iCCA from HCC are reported to be 68.8%–93.5% and 86.2%–97.7%, respectively [41–44]. Notably, recent evidence suggested that gadolinium ethoxybenzyl diethylenetriamine penta acetic acid-enhanced MRI may be used also to evaluate the liver function allowing the identification of patients at higher risk of post-hepatectomy liver failure, thus adding an important value of the standard diagnostic MRI [45].

iCCA is a well-known glucose avid lesion within the liver and fluorodeoxyglucose positron emission tomography (FDG-PET) is effective in detecting MF tumours >1 cm in diameter but shows difficulties in assessing the presence of infiltrating periductal tumours [46]. Despite FDG-PET limitations associated with false-positive results in patients with biliary inflammation and false-negative scans with mucinous tumours [47, 48], its ability to reveal occult metastatic spread in up to 20%–30% of cases has empowered the utilization of FDG-PET in clinical practice. A meta-analysis confirmed the potential of FDG-PET in the assessment of distant and nodal disease in biliary tract cancer (BTC), leading to a change in management in 15% of patients. However, current international guidelines are not in favour of its routine use in the absence of radiological and clinical suspicion of metastatic spread [27, 49]. Conversely, very few studies have assessed the role of FDG-PET for the evaluation of nodal status exclusively, and no recommendations can be drawn because of conflicting results [50]. Whether to perform FDG-PET or not in clinical practice should also be determined by considering its cost-effectiveness.

Due to the high prognostic impact of positive lymph nodes in patients with large tumour burden or at increased surgical risk, preoperative endoscopic ultrasound with fine needle aspiration cytology may be considered to drive clinical decisions.

### Tissue diagnosis and other interventional procedure

Percutaneous biopsy for patients with suspicious or typical imaging findings of iCCA is rarely indicated if a patient is eligible for complete resection [51]. However, in advanced disease not suitable for surgery with difficult differentiation between iCCA, HCC, or metastatic lesions, guidelines suggest its usage. In order to choose the right drug regimen, it is very important to confirm the histological type of the tumour and assess the tumour molecular profile [52]. However, when not necessary, biopsy should be avoided also of the risk of complications, like bleeding and peritoneal dissemination. A meta-analysis showed that the incidence of post-biopsy seeding was 2.7% [53].

Routine staging laparoscopy is not indicated. In patients with iCCA and multifocal disease, high CA 19.9 levels, questionable vascular invasion, or suspicion of peritoneal disease, it could be done to definitively rule out resectability; in this regard, the use of laparoscopic ultrasound may help in identifying intrahepatic metastasis or extensive vascular invasion, undetected by other diagnostic tools [54].

### Screening program

Regarding screening programs, patients with known risk factors may need regular screening using liver function tests, tumour markers, and abdominal ultrasonography. In patients infected with liver flukes, abdominal ultrasound surveillance at 6-monthly intervals is recommended [7, 17, 55]. A retrospective study conducted by the Mayo Clinic showed the benefit of a surveillance programme with annual imaging (abdominal ultrasound or MRI) plus CA 19.9 for patients with PSC [56].

## Current international guideline

Different international guidelines have thrown light on the increasing incidence of iCCA worldwide, stressing the need for a precise *Ad hoc* treatment algorithm. The latest European Association for the Study of the Liver and International Liver Cancer Association (EASL-ILCA) Clinical Practice Guidelines on the management of iCCA [17], released in 2023, together with the 2022 European Society for Medical Oncology (ESMO) clinical practice guidelines for diagnosis, treatment, and follow-up on BTC [11], and the 2023 version of National Comprehensive Cancer Network (NCCN) guidelines on BTC [57], dictate clinical decisions on the management of iCCA in Western countries.

In the meantime, looking at Eastern countries, the Liver Cancer Study Group of Japan published in 2022 the first version of clinical practice guidelines for iCCA [15]. International experts gathered to draft dedicated evidence-based guidelines for physicians involved in the diagnosis, prognostic, and therapeutic management of iCCA.

All of them agreed on the potential curative role of R0 resection for cholangiocarcinoma. Neoadjuvant treatments are not accepted as viable options in the case of resectable iCCA: upfront surgical resection remains the mainstay of treatment to achieve a curative intent. Neoadjuvant systemic chemotherapy can only be considered in patients with technically challenging but resectable disease if a high risk of R1-margin is predicted. After surgical resection, the Japanese guidelines recommend that adjuvant chemotherapy ‘may be considered’. This recommendation is notably weaker than those in the NCCN guidelines (preferred), and the ASCO clinical practice guideline (patients should be offered adjuvant capecitabine), which was established following the results of the BILCAP trial [58].

In the case of unresectable iCCA, the standard of care is systemic treatment: Japanese guidelines recommend gemcitabine with cisplatin, with or without S-1 [59]. In Western countries, there is wide agreement among panels of experts on the use of durvalumab (a type of immunotherapy) in addition to gemcitabine with cisplatin in first-line systemic therapies, especially after strong results obtained from the TOPAZ-1 trial [60]. The Japanese guideline includes no recommendation for patients with genomic alterations in FGFR or IDH1. On the contrary, Western guidelines suggest molecular profiling at the time of diagnosis in cases of advanced disease to use novel target therapies in case of progression on first-line treatment.

**Figure 2.**
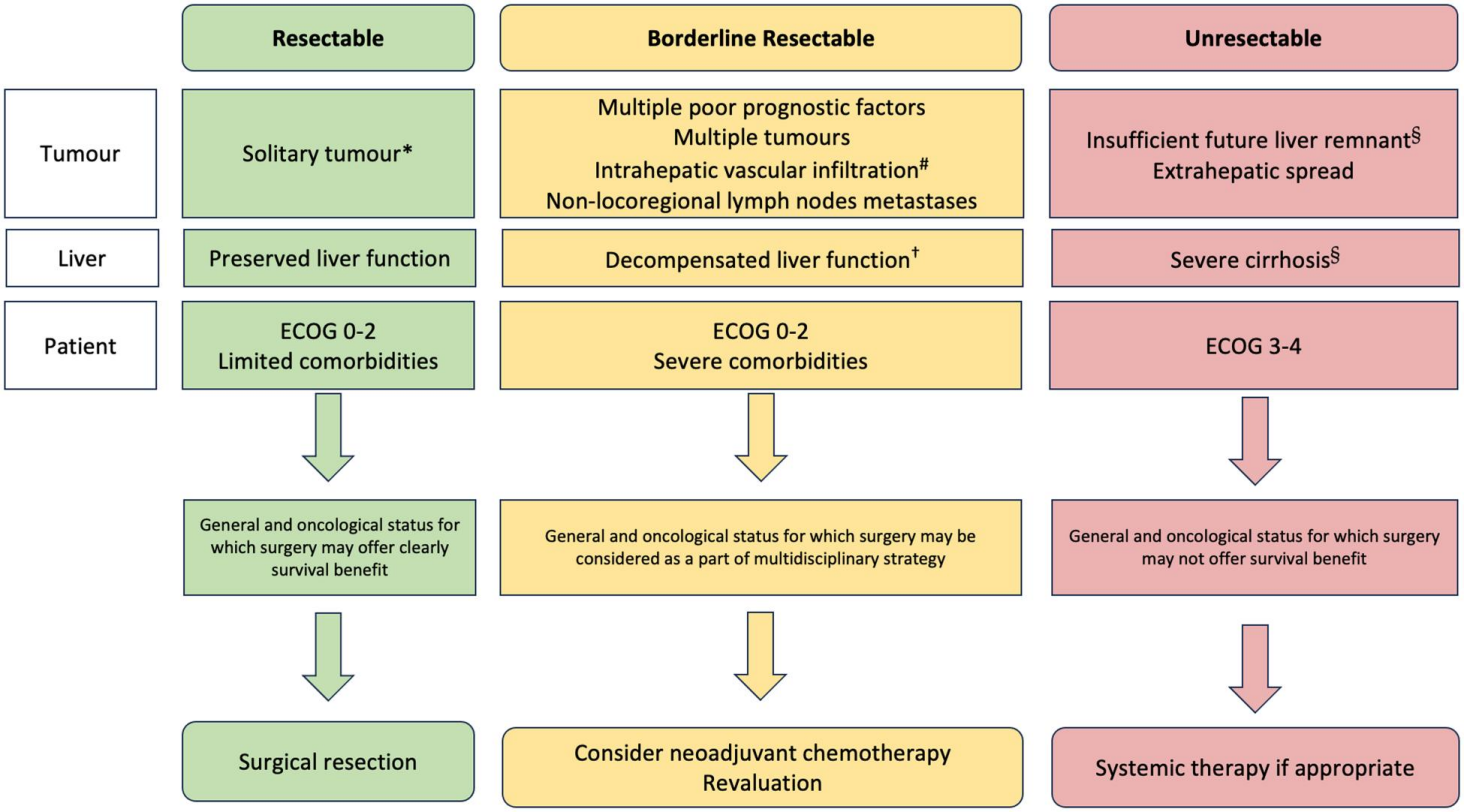
Treatment algorithm for intrahepatic cholangiocarcinoma (iCCA). A traffic-light colour code defines the current approach for iCCA patients. *Also, in the presence of a single poor prognostic factors, such as elevated CA19.9 and/or CEA, size >75 mm, satellite nodules, possible R1v status, regional lymph node metastases (hepatic hilum or level 12). ^#^Impossibility to achieve an R0/R1v resection preserving an adequate future liver remnant. ^†^Consider severity of cirrhosis, level of portal hypertension, and cirrhosis-related complications. ^§^Liver transplantation can be considered in very selected cases in the context of clinical studies. CA19.9 = carbohydrate antigen 19-9, CEA = carcinoembryonic antigen.

Although the incidence of iCCA is gradually increasing worldwide, the number of patients deemed to be resectable is limited. For this reason, clinical cases should be discussed in tertiary centres within multidisciplinary teams to offer the patient the best chance of treatment according to age, comorbidities, local experience, current international guidelines, and stage of disease with risk factors (Figure 2).

## Preoperative chemotherapy

**Figure 3.**
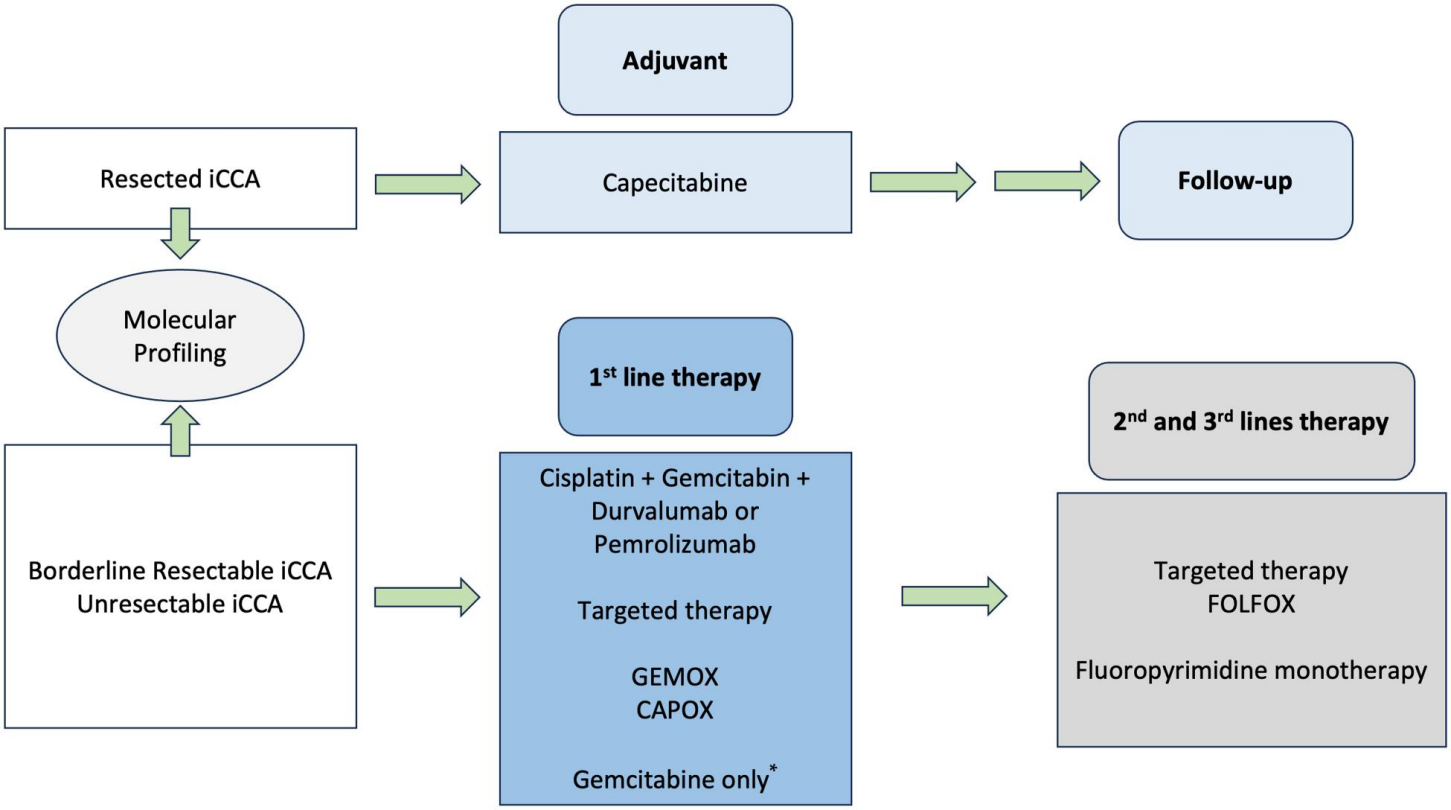
Systemic therapy for intrahepatic cholangiocarcinoma (iCCA). The figure details the current systemic regimens in iCCA patients. *To be considered in frail patients.

Figure 3 details the current standard of care of systemic therapy for iCCA. There is no evidence supporting the use of routine neoadjuvant systemic chemotherapy over upfront resection in patients with resectable iCCA. No randomized studies were identified comparing neoadjuvant chemotherapy followed by surgery vs resection alone [61–63].

However, to further improve treatment outcomes, neoadjuvant chemotherapy should be considered in more advanced cases, such as patients with lymph node metastases (LNMs), intrahepatic metastasis and/or vascular invasion, or high tumour marker [64]. However, the inclusion of resectable patients leaves the possibility that cases become unresectable after neoadjuvant chemotherapy, losing the chance of gaining advantage from the only recognized curative treatment, which is R0-resection. Arguments in favour of systemic preoperative treatment include the need to decrease the high recurrence rates, considering the high incidence of R1-resection, due to the frequent wide extension of the disease. Furthermore, preoperative systemic chemotherapy can be considered a ‘test of time’ for aggressive disease: some argue that progression during neoadjuvant chemotherapy predicts future dismal outcomes with resection, allowing avoidance of demanding and probably worthless extended surgical procedures that are not free from risks of postoperative complications. Administration of chemotherapy in patients with unresectable disease has been reported to result in a reduction in tumour size and vascular invasion, and the disappearance of abnormal uptake in regional and distant lymph nodes on FDG-PET followed by conversion to resectable disease [15, 65, 66]. Although there is a lack of randomized prospective studies supporting neoadjuvant chemotherapy, its use in downsizing and downstaging tumours in the preoperative setting is getting interest, and some promising results have been shown by retrospective series of patients with iCCA, preserved liver function, and no cholestasis. As is broadly known, however, retrospective series, by their nature, lack a comparator arm to gauge the magnitude of benefit. In a phase II trial, Maithel *et al.* [67] evaluated the GAP regimen (gemcitabine, cisplatin, and nab-paclitaxel) and found it to be both safe and feasible without adversely impacting perioperative outcomes. In this trial, patients were classified as high-risk based on various tumour-specific factors, including T-stage Ib or greater, solitary lesions larger than 5 cm, multifocal tumours, satellite lesions confined to the same liver lobe as the dominant lesion, major vascular invasion (but technically resectable), and suspicious or involved regional lymph nodes. The authors concluded that the neoadjuvant GAP regimen was safe and feasible without negatively affecting perioperative outcomes. Therefore, it may be reasonable to consider neoadjuvant therapy for high-risk patients, ideally as part of a clinical trial (Table 1).

**Table 1.** Ongoing clinical trials on preoperative approaches for iCCA

Trial ID	Institution	Enrolment (estimated) patients	Treatment arm	Primary outcome
NCT04961788	Shangai Zhongshan Hospital	30	Toripalimab+Gemox	12-month ORR
NCT06208462	Nanjing Medical University	33	HAIC (GEMOX)+adebrelimab+lenvatinib	12-month ORR
NCT06050252	National Cancer Institute	27	Gem-Cis+durvalumab	Proportion of patients who complete four cycles of neoadjuvant therapy followed by surgical resection
NCT04989218	University of Alabama at Birmingham	20	Durvalumab, tremelimumab+platinum-based chemotherapy	12-week (four cycles) ORR
NCT04523402	Eastern Hepatobiliary Surgery Hospital	100	Oxaliplatin+gemcitabine+resection vs resection only	24-month PFS
NCT0555757	Zhejiang Cancer Hospital	20	Tislelizumab combined with GEMOX regime	R0 resection rate, objective response rate
NCT06017297	Georgetown University	28	Tremelimumab+durvalumab+Gem/​Cis	Rate of conversion from unresectable to resectable
NCT05672537	Tianjin Medical University Cancer Institute and Hospital	70	Gem-Cis+durvalumab+resection vs only surgical treatment	12-month PFS
NCT05290116	Sun Yat-Sen University	17	Hepatic arterial infusion chemotherapy+tislelizumab+apatinib	12-month ORR
NCT04954781	Fudan University	25	Transarterial chemoembolization+tislelizumab	24-month ORR
NCT04546828	Samsung Medical Center	34	Gemcitabine, Cisplatin, Nab-Paclitaxel	16-week increased rate of R0 resection
NCT04669496	Shangai Zhongshan Hospital	178	Gemox+lenvatinib, toripalimab vs only adjuvant capecitabine	24-month EFS
NCT04523402	Eastern Hepatobiliary Surgery Hospital	100	Gemox vs No neoadjuvant	24-month EFS

iCCA = intrahepatic cholangiocarcinoma, ORR = overall response rate, PFS = progression-free survival, EFS = event-free survival.

Another retrospective analysis from Japan reported a conversion rate of 36.4% after chemotherapy (gemcitabine monotherapy) for unresectable BTC [65]. The reasons for not performing initial resection in patients with iCCA were extensive vascular invasion in three patients and insufficient expected residual liver volume (RLV) after resection in one patient. Twenty-two patients with unresectable iCCA who received neoadjuvant gemcitabine were downstaged to resection and achieved a 5-year overall survival (OS) of 45%.

The largest retrospective series is a French study focused on patients with initially unresectable iCCA [61]. Of 186 patients, 74 received chemotherapy with disparate regimens and 39 of those 74 (53%) underwent resection following chemotherapy. The median OS was 24.1 months, which was like that observed in patients who had upfront resectable disease (median OS: 25.7 months).

However, either the Japanese and French studies were retrospective, and they offered heterogenous treatments that does not allow to draw valid conclusions on preoperative systemic chemotherapy in iCCA.

Three propensity score-matched analyses have been reported, all using data from the National Cancer Database. Yadav *et al.* [68] matched 278 patients with stage I-III iCCA who received neoadjuvant chemotherapy (203 of whom had iCCA) with 700 patients (487 iCCA) who underwent surgery followed by adjuvant therapy. Patients receiving neoadjuvant chemotherapy had an improved OS (median 40.3 months vs 32.8 months) and were more likely to have an R0 resection (71.2% vs 61.6%). The survival advantage did not change in the subgroup of patients with iCCA. Another analysis, covering an additional 2 years (2006–2016) and using the same database, showed that neoadjuvant treatment was more likely to be used in patients with radiological evidence of lymph node involvement or T2/T3 disease. After propensity matching for these parameters, they found a 23% reduction in risk of death from neoadjuvant treatment [69].

Platinum-based chemotherapy is the cornerstone of modern systemic therapy; however, its use is often limited by severe cytotoxic side effects and a low therapeutic index. Targeted molecular therapy is a growing area of interest across nearly all oncology fields, and cholangiocarcinoma is no exception. Next-generation sequencing has facilitated the identification of specific genetic mutations driving cholangiocarcinogenesis. By blocking key biological pathways or mutant proteins, targeted therapies can arrest tumour growth and promote regression. Immunotherapeutic strategies for treating iCCA have been enabled by a deeper understanding of intratumoural heterogeneity. Adoptive cell therapy and immune checkpoint inhibitors (ICIs) underscore immunotherapy’s role in enhancing natural anti-tumour immune responses, enabling the generation of anti-tumour memory and prolonged tumour destruction [70]. ICIs targeting programmed death 1 (PD-1), programmed death-ligand 1 (PD-L1), and cytotoxic T-lymphocyte antigen-4 (CTLA-4) are pivotal in countering the tumour-tolerant microenvironment in iCCA [71]. Immunotherapies targeting PD-1 and its ligand are gaining increasing attention. Approximately 10% of individuals with iCCA have either microsatellite instability or lack mismatch repair proteins, making them suitable candidates for immunotherapy [72]. Based on promising results from PD-L1 blockade, the United States Food and Drug Administration (FDA) approved pembrolizumab for microsatellite instability-high/mismatch repair-deficient tumours in 2020 [72]. In a retrospective review with propensity score matching by Rizzo *et al.* [73], patients with advanced BTCs who received anti-PD-1 therapy in addition to chemotherapy had longer progression-free survival (PFS) than those receiving chemotherapy alone. In a case report by Zhang *et al.* [74], a 38-year-old female with iCCA achieved an R0 resection and extended survival following neoadjuvant PD-1 blockade and tyrosine kinase inhibitor therapy. The DEBATE Trial (Neoadjuvant Gemcitabine plus Cisplatin with or without Durvalumab in resectable BTC) is currently recruiting patients; its results may provide future guidance for PD-L1-targeted treatments in cholangiocarcinoma.

Additionally, the possible role of hepatic arterial infusion (HAI) chemotherapy in combination with systemic chemotherapy has also been examined. Encouraging data come from a retrospective series [63], where 104 patients with iCCA confined to the liver received systemic chemotherapy combined with HAI (78 patients) or systemic chemotherapy alone (26 patients). The group receiving combined therapy had a superior OS (30.8 months vs 18.4 months). Furthermore, eight patients with initially unresectable iCCA were able to undergo surgery following a response to treatment, achieving a median OS of 37 months.

Although transarterial chemoembolization (TACE) has proven to be effective for disease control in the advanced setting, few reports are available on its role in the neoadjuvant setting, all with disappointing response rates, perhaps as a consequence of the hypovascular nature of iCCA, characterized by extensive fibrosis and a predominantly non-arterial blood supply [75].

Finally, neoadjuvant chemotherapy has also been applied to liver transplantation instead of resection. Despite these encouraging initial results, there is a need for controlled studies that pay attention to standardization of outcomes and duration of therapy.

In any case, the implementation of neoadjuvant protocols in the treatment of iCCA should always be discussed by multidisciplinary boards [17] (Figure 2).

## Surgical resection for iCCA

As mentioned above, R0 liver resection is the only curative treatment for iCCA: it is strongly recommended by several guidelines and consensus statements as it is associated with better clinical outcomes than R1/R2 resections [54, 76–78]. Regrettably, most patients are unresectable because of late diagnosis: according to the Surveillance, Epidemiology, and End Results (SEER) database, only 15% of patients with iCCA diagnosed between 1983 and 2010 underwent resection [5]. Even though only a minority of patients with iCCA are candidates for liver resection (LR), the number of hepatectomies for iCCA is increasing worldwide. This is related to technical advances in the field of hepatobiliary surgery and improved perioperative management, which become evident when comparing the results of liver surgery in high-volume referral centres vs low volume centres [79]. Yet, in high-volume hepatobiliary centres, surgical mortality after iCCA resection is reported to be lower than 5% [80]. The median OS after LR is reported to be 40 months, with a 5-year OS of 25%–40% [81–83].

Tumour size and location, the presence of satellite nodules, the number of tumours, and the presence or absence of LNMs are known to be important prognostic factors. The quantity and quality of liver parenchyma that remains following tumour excision are the two key factors that determine resectability with clear surgical margins.

### Tumour size and location

A solitary tumour with no LNMs is the best indication for LR. In cases of relatively small and peripheral lesions, non-anatomical or anatomical LR are usually performed. A report has noted that patients with a tumour measuring ≤2 cm with no LNMs (N0), no portal vein invasion, and no bile duct invasion presented a 100% 5-year survival rate, and thus, complete cure could be expected from surgical resection [84]. However, Wang *et al.* [85] found that patients with iCCA benefited from anatomical in OS and DFS after propensity score-matching analysis, indicating complete removal of tumour-bearing segments plays a significant role in improving the survival outcomes. On the other hand, larger multisegmental tumours present a significant risk of recurrence and they often require more complex anatomical LR. A pivotal study, analysing data on 702 consecutive patients using a propensity score-matching analysis, concluded that anatomical resection was associated with better survival compared to non-anatomical resection for stage IB or II iCCA without vascular invasion [82].

Controversies persist in the surgical community about the definitions of tumour margins and anatomical resection that lead to difficult interpretation of these results [86]. The location of the tumour, especially its relationship to intrahepatic vascular and biliary structures for centrally located lesions, is essential for current surgical planning to obtain an R0 resection margin. Indeed, the application of R1-vascular hepatectomy in iCCA patients should be limited to those patients otherwise considered unresectable [87]. It is essential to assess the relationship of the lesion with the first- and second-order portal and biliary branches, as well as with hepatic veins. Well-defined contraindications to liver resections include bilateral involvement of second-order biliary branches, unilateral liver atrophy with contralateral biliary or vascular involvement (either portal or arterial), and unilateral biliary involvement linked to contralateral vascular infiltration (either portal or arterial). LR associated with biliary tree resection is indicated in tumours invading the ductal bifurcation and/or the main hepatic duct, as required in 20%–30% of LR for iCCA [80, 88]. The presence of vascular/bile duct invasion is associated with poor prognosis but does not indicate unresectable iCCA. Patients undergoing major resections, including those involving the inferior vena cava and portal vein, had similar results to those undergoing conventional resections. This suggests that major vascular resections can be considered, without major impact on clinical outcomes, if R0 resection is achievable [89, 90].

### Multifocal disease

Multifocal iCCA is seen in nearly 50% of patients [91, 92], and the role of surgery for these patients remains debatable. Compared to patients with a single lesion, patients with multifocal iCCA are more likely to experience tumour recurrence and death following LR [81, 92]. A study compared patients with multifocal disease who underwent surgical resection to those with single tumours. The median survival of those with multifocal disease was 21.2 months for patients with two tumours and 15.3 months for those with three or more, while it was 43.2 months for those with a single tumour [93]. Another study from Spolverato *et al.* [91] demonstrated similar results, with a 5-year OS rate of 30.5% for patients resected with single tumours and 18.7% for those with multifocal disease. The 5-year survival rate decreases with an increasing number of tumours: 46.8% for a single tumour, 33.6% for two tumours, and 11.1% for three tumours [94].

### Resectability and RLV

A precise assessment of RLV is essential to prevent postoperative liver failure. The quality of the liver remnant is also important because long-term cholestasis-related atrophy and fibrosis, or steatosis and fibrosis, might hinder the regeneration of the remaining liver following resection. In patients with a normal liver, 30% of RLV is sufficient to prevent liver failure in the postoperative phase, while more than 40% of RLV is usually necessary in patients with chronic liver diseases [95]. Since postoperative liver failure is the most frequent cause of mortality after extended hepatectomy, strategies to enable this surgical procedure in otherwise resectable tumours have been explored. When the RLV is insufficient, the most common method used on patients undergoing major LR is currently portal vein embolization (PVE). Indeed, a systematic review showed how PVE resulted in a marked decrease in liver failure and 90-day mortality in patients with iCCA undergoing major liver resection [96]. Therefore, guidelines suggest PVE in patients without jaundice or cirrhosis who are undergoing hepatic resection with insufficient RLV [54, 57, 76, 77]. Also, in iCCA patients with insufficient future liver remnant (FLR) (i.e. below 40%) preoperative biliary drainage of the FLR could be required prior to PVE in cases of biliary obstruction. For patients with an FLR volume above 50%, the risk of cholangitis and related mortality after drainage does not justify the potential benefit of biliary decompression. The indication for preoperative biliary drainage must therefore be cautiously evaluated by a hepatobiliary multidisciplinary team [51]. Associating liver partition and portal vein ligation for staged hepatectomy (ALPPS) is an accelerated procedure that may allow for faster and enhanced hypertrophy of the liver remnant (up to 70%) compared to conventional portal embolization in patients with very low FLR volume, those expected to have insufficient regeneration after PVE, or those who may be at a high risk of tumour progression (possibly impairing the chances of surgical resection during the regeneration phase) [97]. The procedure may be considered a modification of the two-staged liver resection, combining two established surgical techniques: right portal vein ligation and *in situ* splitting of the liver. However, an Italian multicentre study [98] and a recent case-control study conducted in the ALPPS International Registry [99] showed a high postoperative mortality rate (40%–44%) in the setting of iCCA, and therefore ALPPS should be reserved for experienced centres in highly selected patients [54]. A more recent adaptation of the original method is partial ALPPS which has demonstrated comparable effects on hypertrophy with a considerable decrease in perioperative morbidity when only 50% of the liver parenchyma was transected [100]. Another potential procedure to enhance hypertrophy of FLR is simultaneous or sequential occlusion of the hepatic vein(s) in addition to portal vein embolization (PVE+HVE). Kobayashi *et al.* [101] reported their experiences of simultaneous hepatic vein embolization with PVE, termed ‘liver venous deprivation’ (LVD). In this study, 21 patients underwent LVD leading to a superior degree of hypertrophy and kinetic growth rate compared with 39 patients undergoing PVE alone, without an increased incidence of per-procedural complications. Despite the faster rate of hypertrophy, whether HVE should be performed routinely or selectively after the failure of PVE remains unclear. However, given the encouraging results, LVD could be a potential choice of preoperative hemodynamic modulation, especially for patients presenting with a very small FLR before planned extended hepatectomy [102, 103].

### Lymph node metastases

About one-third of patients undergoing surgical resection of iCCA present LNM, with a 5-year survival rate significantly decreased in these patients [15, 104, 105]. The incidence of LNM in iCCA is reported to be about 30%–53% for the MF type and more than 60% for the PI or MF+PI types. On the other hand, the intraductal-growth type is rarely associated with LNM [105–109].

LNM commonly occurs in nodes in the hepatoduodenal ligament, around the common hepatic artery, and behind the pancreatic head. Metastasis to the LNs of the gastric lesser curvature can also occur in patients with iCCA in the left liver. There is an ongoing discussion on the significance of hilar lymphadenectomy (LND) in iCCA. NCCN and ILCA guidelines indicate that an LND is considered adequate when a minimum of six nodes are harvested [110].

Despite these recommendations, the use of LND in the surgical management of iCCA remains relatively low, with only about 30%–50% of patients undergoing LND, even at high-volume centres [81, 111]. Some surgeons choose not to perform LND for iCCA due to a perceived lack of therapeutic benefit. While LND in clinically node-positive patients (cN+) has become more widely accepted and performed, the need for LND in patients without preoperative evidence of LN metastases (clinically node-negative, cN0) is still under debate. In a retrospective cohort study of 706 patients who underwent curative surgery for cN0 iCCA, Sposito *et al.* [112] demonstrated that adequate LND provided better survival outcomes for patients with cN0 iCCA who were found to be node-positive at pathology, supporting the routine use of adequate LND in cN0 iCCA cases. One potential benefit of prophylactic LND is the prevention of nodal recurrence in the hepatoduodenal ligament, as LNM in the perihepatic area may lead to early obstructive jaundice and shorten post-recurrence survival.

With the advent of more effective adjuvant protocols, it is expected that precise nodal staging will become of paramount importance soon for directing tailored therapeutic options.

### Surgical approach

Regarding the surgical approach, laparoscopic LR is associated with better short-term outcomes, improved pain management, and shorter hospital stays [113]. A propensity-matched cohort study comparing laparoscopic to open LR showed similar outcomes between groups, with a median disease-free survival of 33 months for the laparoscopic group and 36 months for the open surgery group [99, 114]. Although the use of robotic technology has not been specifically evaluated for iCCA, patients undergoing robotic hepatectomy for various hepatic tumour types have shown promising outcomes [115]. Robotic surgery may have additional advantages, such as surgeon comfort, shorter hospital stays, and better short-term outcomes. Moreover, it is likely that the robotic approach will also facilitate portal LND [116–118].

However, the open approach still represents the standard of care in iCCA surgery also considering the relatively high proportion of patients who present with high intrahepatic tumour burden.

### Recurrent disease

The pattern of recurrence in iCCA is unpredictable. While intrahepatic recurrences are the most frequent (occurring in around 60% of cases) and often present in cases with multiple sites of recurrence, extrahepatic recurrences also occur, particularly in patients with lymph node involvement [80, 119, 120]. Aggressive surgical treatment is feasible for recurrent iCCA. Recently, nomograms derived from multi-institutional analyses have been developed to improve prognostic classification for iCCA patients undergoing resection. These tools may help identify patients who are more likely to experience recurrence and could benefit from adjuvant and/or neoadjuvant treatments. The nomograms incorporate clinicopathological and laboratory data, including tumour size, multifocality, tumour burden score, KRAS status, vascular invasion, nodal involvement, presence of cirrhosis, and preoperative serum tumour marker levels [120, 121].

However, these nomograms are limited by small sample sizes, institutional practice variations, and selection bias, as they include only patients who underwent resection. Although nomograms generate intellectual interest, they are not yet widely used in clinical decision-making for iCCA patients.

### The role of adjuvant chemotherapy

Most studies on adjuvant therapy for iCCA are retrospective in nature and can be roughly divided into two groups: studies on BTC, which include extrahepatic cholangiocarcinoma and gallbladder cancer, and studies focusing solely on iCCA. Stratified analysis for iCCA is not included in the majority of BTC studies. A meta-analysis showed that adjuvant chemotherapy is associated with improved OS and should be offered to patients with iCCA after surgery, especially those with advanced disease [122].

The results of the long-term outcomes of the BILCAP study were published: the median OS was 49.6 months in the capecitabine group, compared to 36.1 months in the observation group, without differences depending on the site of CCA (iCCA vs dCCA) [58]; the study was negative for the primary endpoint on intention-to-treat analysis but positive on per-protocol analysis. An American Society of Clinical Oncology (ASCO) expert panel conducted a systematic review to guide decision-making for patients with resected biliary cancer and concluded that adjuvant capecitabine should be offered [113].

In a clinical study designed to examine whether gemcitabine plus oxaliplatin combination therapy would prolong recurrence-free survival compared with surgery alone (PRODIGE 12-ACCORD 18 study), the superiority of the combination therapy was not demonstrated [123]. In the study, about 45% of patients had iCCA, and even stratified analysis showed no superiority of this adjuvant chemotherapy.

Another fluoropyrimidine, S-1, has been evaluated in the Japanese ASCOT study: 440 patients were randomized to either surgery alone (222 patients) or adjuvant S-1 (218 patients). The study revealed an advantage in terms of OS for the iCCA subgroup [124].

When considered collectively, the most trustworthy evidence is that adjuvant chemotherapy with capecitabine alone demonstrated a survival benefit in BTC, including iCCA, in only the per-protocol analysis of a prospective comparative study (Table 2).

**Table 2.** Ongoing clinical trials on adjuvant therapies for iCCA

Trial ID	Institution	Enrolment (estimated) patients	Treatment arm	Primary outcome
NCT06406816	Meng Chao Hepatobiliary Hospital of Fujian Medical University	10	Neoantigen vaccine plus capecitabine for the treatment of high-risk recurrent intrahepatic cholangiocarcinoma	Safety endpoint
				12-month OS
				12-month PFS
NCT04782804	Fudan University	30	Capecitabine+PD-1 antibody (Tislelizumab) vs capecitabine alone	24-month PFS
				5-year OS
NCT06109779	Astra Zeneca	750	Rilvegostomig+capecitabine or gemcitabine/cisplatin or S-1 vs adjuvant chemotherapy alone	5-year PFS

iCCA=intrahepatic cholangiocarcinoma, OS=overall survival, PFS=progression-free survival, PD-1=programmed death 1.

### Locoregional treatment

While surgical treatment represents the standard of care for resectable iCCA, small lesions of less than 3 cm located centrally in the liver may be treated with thermal ablation approaches, such as radiofrequency ablation and microwave ablation, especially in patients at high surgical risk, such as those with cirrhosis or multiple comorbidities. The main risk of thermal ablation is the rate of local or systemic recurrence, possibly due to the lack of LND [51]. Nowadays, locoregional therapies are mainly applied in patients with unresectable disease. Patients who are not suitable for systemic therapies due to impaired liver function or other comorbidities may benefit from locoregional treatments to control liver disease. It is well known that most patients die from tumour burden: progressive disease in the liver eventually causes obstruction and liver failure.

Other approaches for advanced iCCA include embolization therapies, such as TACE with or without drug-eluting beads and yttrium-90-labelled selective internal radiation therapy [125–127]. Additionally, stereotactic radiotherapy may be considered for unresectable iCCA with tumour diameter <5 cm in the absence of metastases [15]. Combination therapy could also result in a more effective strategy than single-agent therapy. As shown in the prospective study by Kiefer *et al.* [128] survival was improved in those patients receiving locoregional therapy and concomitant systemic chemotherapy, with an OS up to 28 months for treated iCCA. The combination of TACE and systemic therapy revealed a survival benefit of up to 7 months over best supportive care [129].

HAI has also showed interesting results. Using an implanted, subcutaneous pump, patients with iCCA have been treated with continuous hepatic artery infusion of fluxoridine (FUDR) [130], 5-fluorouracil and oxaliplatin [131], gemcitabine [132], epirubicin, and cisplatin [133]. A study using HAI with FUDR combined with systemic gemcitabine and oxaliplatin confirmed a response rate of 46% with a 2-year OS of 53% [134], although 10% of patients developed significant complications like portal hypertension, gastroduodenal artery aneurysm, catheter displacement, and jaundice. HAI remains an attractive concept to potentially downstage large, localized iCCA to achieve resection or to prevent intrahepatic progression and appears to be more effective than TACE or intraarterial radioembolization in both local control of the disease and OS [75]. However, liver function must be carefully examined before treatment as underlying cirrhosis should be considered a contraindication due to the increased risk of liver-related complications [134].

### Transplantation for iCCA

In many centres, iCCA still represents a contraindication for liver transplant due to high recurrence rates, with microvascular invasion and poor tumour differentiation being associated with tumour recurrence [135, 136]. While liver transplant remains contraindicated for large iCCA, the scenario is likely to change for small iCCA. Available evidence comes from observational studies; however, ongoing prospective studies are investigating liver transplantation for early iCCA, arising in the context of cirrhosis. In a retrospective, multicentre study, Sapisochin *et al.* [137] demonstrated that patients with a single iCCA less than 2 cm (very early iCCA) had a 5-year OS of 65% and a 5-year cumulative incidence of recurrence of 18%. The outcomes in the very early iCCA group were superior to those in patients with advanced iCCA, who achieved a disappointing 5-year OS of 45% and a 5-year cumulative incidence of recurrence of 65%. These results suggest that liver transplantation might be an option for patients with very early iCCA who are not candidates for liver resection due to advanced cirrhosis. These promising results need further investigation to confirm the effectiveness of liver transplantation in this setting.

Liver transplantation has also been examined for locally advanced, liver-only, unresectable iCCA. Past results previously showed dismal outcomes. The most relevant study is the one from McMillan *et al.* [138]. Patients undergoing liver transplantation were required to demonstrate disease stability for 6 months on neoadjuvant therapy with no extrahepatic disease. During the study period, 32 patients were listed for liver transplantation and 18 patients underwent the procedure. The study showed encouraging results with 1-, 3-, and 5-year OS rates of 100%, 71%, and 57%, respectively. Recurrence occurred in seven patients and was treated with systemic therapy and resection. Despite the promising results, further studies must be conducted to identify more precise criteria for liver transplantation in this context. Machine perfusion and living donor liver transplantation could be useful tools to enlarge the pool of donors for innovative indications like iCCA [43].

### Targeted therapies and immunotherapy for advanced disease and disease recurrence

Given the identification of several clinically significant molecular changes in iCCA, numerous studies have assessed the application of targeted treatments, especially for advanced disease (Table 3). In the most recent NCCN guidelines, molecular testing is now recommended for patients with unresectable or metastatic BTC [78]. Due to the high rate of recurrence, we also recommend considering genetic testing at the time of resection. Key targets include isocitrate dehydrogenase-1 (IDH1), fibroblast growth factor receptor-2 (FGFR2), human epidermal growth factor receptor-2 (HER2/ERBB2), proto-oncogene B-Raf (BRAF), high tumour mutational burden (TMB-H), high microsatellite instability or mismatch repair deficiency (MSI-H/dMMR), neurotrophic tyrosine receptor kinase (NTRK) fusions, and rearranged during transfection (RET) fusions. Some of these drugs have been FDA-approved for advanced ICC, with ongoing research.

**Table 3.** Targeted therapies and most common targets of interest

Target	Estimated prevalence	Therapy
IDH1	10%–20%	Ivosidenib
FGFR2	9%–15%	Futibatinib, pemigatinib, infigratinib
HER2	5%–20%	Transtuzumab, pertuzumab, neratinib, lapartinib
BRAFV600E	<5%	Dabrafenib+trametinib
TMB-H	<5%	Nivolumab+ipilimumab
MSI-H/dMMR	<5%	Pembrolizumab
NTRK	<1%	Entrectinib, larotrectinib
RET	<1%	Selpercatinib, pralsetinib

IDH1=isocitrate dehydrogenase-1, FGFR2=fibroblast growth factor receptor-2, HER2=human epidermal growth factor receptor-2, TMB-H=high tumour mutational burden, MSI-H/dMMR=high microsatellite instability, or mismatch repair deficiency, NTRK=neurotrophic tyrosine receptor kinase, RET=rearranged during transfection.

The two mutations more commonly found in iCCA than other BTCs are IDH1 mutations and FGFR2 fusions or rearrangements. Approximately 10%–20% of ICC cases present IDH1 mutations, which can be targeted with ivosidenib [139]. In the ClarIDHy phase III multicentre trial, 185 patients with IDH1-mutated cholangiocarcinoma (91.4% ICC) whose disease had progressed on standard chemotherapy were randomized to receive either ivosidenib or a placebo [140]. After a median follow-up of 6.9 months, ivosidenib demonstrated an improved PFS over the placebo, with a median PFS of 2.7 months vs 1.4 months. Adjusting for crossover between arms, the median OS was 10.3 months with ivosidenib compared to 5.1 months for the placebo [141].

FGFR2 mutations, identified in 9%–15% of iCCA cases, can be targeted with futibatinib, pemigatinib, and infigratinib [138]. Currently, evidence for these agents is limited to phase II trials in previously treated advanced cholangiocarcinoma; however, the promising results suggest clinical benefit with manageable adverse effects.

Although more prevalent in gallbladder carcinoma, HER2 amplification or overexpression is observed in an estimated 5%–20% of cholangiocarcinomas [142]. Recent phase II trials have shown efficacy with HER2-targeted therapies, such as trastuzumab and pertuzumab, achieving a 23% objective response rate (ORR) in patients with metastatic BTC with HER2 overexpression or amplification [143].

RET and NTRK fusions are rare in BTC, occurring in fewer than 1% of patients. Larotrectinib and entrectinib are approved for patients with NTRK fusions, with phase I/II basket trials showing ORRs over 50%, though these studies included only three cholangiocarcinoma patients [144, 145]. Similarly, the RET inhibitors selpercatinib and pralsetinib demonstrated over 40% efficacy in phase I/II basket trials, although each study included three or fewer patients with cholangiocarcinoma [146, 147].

The remaining targets are present in less than 5% of BTCs [148]. For instance, BRAF V600E mutations have been targeted with the combination of dabrafenib and trametinib. In the phase II ROAR basket trial, this oral combination was evaluated in 43 advanced cholangiocarcinoma patients with prior systemic therapy and BRAF V600E mutations, resulting in an ORR of 51% [149].

Current immunotherapy recommendations derive from two phase II trials. Although nivolumab and ipilimumab are more commonly used for advanced melanoma and non-small cell lung cancer, the CheckMate 848 study examined their combination in other solid tumours with TMB-H. Preliminary results showed an ORR of 35.3% among the 68 patients with tissue TMB-H [150]. In the KEYNOTE-158 cohort K study, pembrolizumab was used to treat 321 patients with previously treated non-colorectal MSI-H/dMMR solid tumours, including 22 with cholangiocarcinoma [151]. The ORR for the entire cohort was 30.8%, with an ORR of 40.9% specifically in the cholangiocarcinoma group.

These findings underscore the challenges of building strong evidence for targeted therapy in iCCA due to low patient accrual. However, with the continuous advancements in next-generation sequencing, more actionable targets in iCCA are anticipated in the future.

### Translational research in iCCA

To understand the biological complexity of iCCA, significant efforts have been dedicated to advancing cutting-edge technologies over the past decades. Translational research has played a pivotal role, leading to significant advancements in understanding iCCA pathogenesis and identifying potential therapeutic targets (Table 4).

**Table 4.** Translational research in iCCA

Methodology	Description	References
3D culture method	Patient-derived organoid	[136, 152–154]
	Cholangiocarcinoma-on-chip	[156]
Immunoprofiling	Immune heterogeneity	[157, 158]
	Immune infiltration and patient outcome	[158, 159]
Metabolomic approaches	Metabolites, lipids, and patient prognosis	[164, 165]
	Deregulated metabolic pathway	[162, 163, 166]
Biomarker discovery	Liquid biopsy	[169, 170]
	Tissue biomarker	[171, 172]

iCCA=intrahepatic cholangiocarcinoma.

**Figure 4.**
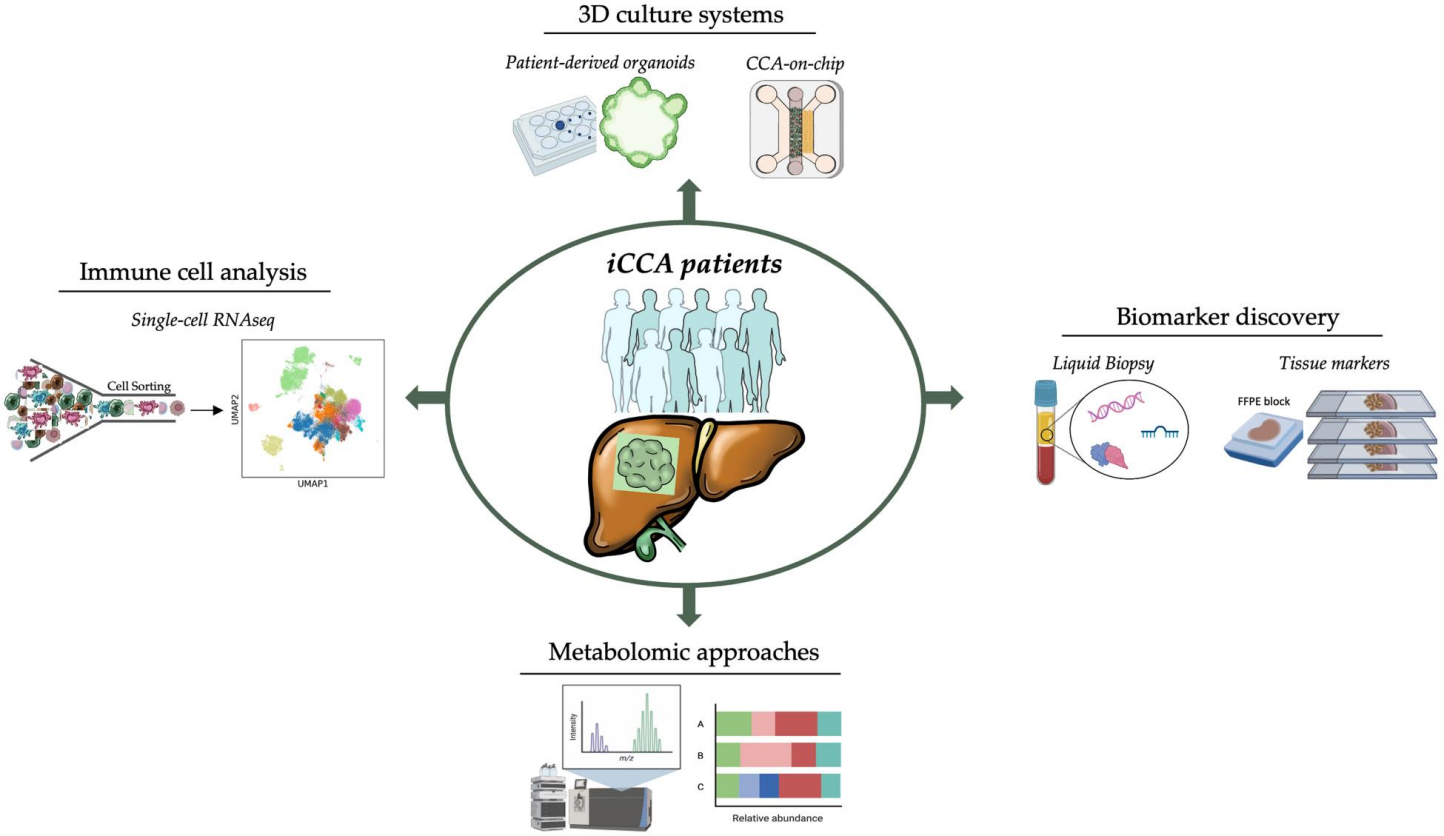
Research approaches used in the laboratory for intrahepatic cholangiocarcinoma. These approaches can be summarized in four areas: (1) 3D culture systems, such as tumouroids, organoids, and chip-systems; (2) biomarker discovery on circulating liquids or tissues; (3) metabolomic approaches; (4) immune-cells system analyses.

Figure 4 details the research approaches currently used in the lab for iCCA studies. Several researchers have focused on enhancing cell culture techniques by establishing 3D culture systems, including patient-derived organoids (PDOs) and the microfluidic platform Cholangiocarcinoma-on-chip (CCA-on-chip), to address the limitations of traditional 2D methods and the animal models. The PDO cultures have been shown to mirror the 3D structure of the original tumour, preserving the transcriptomic alterations, histological characteristics, and marker expression of the patient tumour tissue [136, 152–154]. However, most tumour organoids lack immune and stromal cells, which are essential for tumour growth and immunotherapy response [155]. Conversely, the CCA-on-chip platform integrates microfluidics and tissue engineering to emulate the *in vivo*-like tissue architecture. Indeed, this platform replicates the interactions between different cell types, such as tumour cells, cancer-associated fibroblast, endothelial cells, and immune cells, providing a dynamic and physiologically relevant environment for studying iCCA [156]. Nevertheless, the CCA-on-chip and PDO provide reliable methods for studying tumour biology and facilitating high-throughput drug screening, paving the way for personalized medicine [154–156].

The immune system has been shown to play a critical role in the iCCA development and progression. Recent studies using advanced immunoprofiling techniques, such as single-cell RNA sequencing (scRNA-seq), have revealed that iCCA tumours exhibit a heterogeneous immune infiltrate, which has a significant impact on patient outcomes [157, 158]. High regulatory T-cell infiltration was associated with a worse prognosis, while high levels of CD8+ tumour-infiltrating lymphocytes correlated with improved outcomes and immunotherapy responses [158, 159]. Furthermore, iCCA with high immune checkpoint expression, such as PD-1/PD-L1, may benefit from ICIs [160].

Along this line, another recent study investigated the relevance of systemic inflammation in iCCA patients by considering the combination of blood inflammatory indexes and the iCCA microenvironment suggesting a link between immune-cell infiltration and the prognosis [161]. Yet, understanding the immune milieu and its interactions with iCCA cells offers promising avenues for targeted immunotherapy strategies.

Metabolomic technologies have emerged as a powerful tool in iCCA research, which could help to identify metabolic changes associated with tumorigenesis and iCCA progression [162, 163]. Indeed, metabolomic profiling of iCCA tissues and blood samples revealed altered lipid metabolism and increased glycolytic ability, linked to aberrant mitochondrial activity and deregulated enzymes promoting oncogenic pathways [163–166]. The metabolic shift associated with significantly altered metabolites emphasizes the potential for metabolomics-driven therapy approaches [162, 166].

Biomarker discovery is crucial for enhancing iCCA diagnosis, prognosis, and treatment. Advances in ‘omics’ approaches, including genomic and proteomic technologies, have led to the identification of several potential biomarkers. Liquid biopsies, which analyse circulating tumour DNA (ctDNA), microRNAs, and proteins in the blood, offer an innovative minimally invasive method for monitoring disease progression and treatment response [167, 168]. Studies on ctDNA in iCCA patients have demonstrated that it accurately reflects the tumour’s mutational status, suggesting that its screening could guide potential mutation-based therapeutic interventions [169, 170]. Additionally, several biomarkers identified in tumour tissue are particularly valuable for predicting the prognosis of resected CCAs [171, 172]. This highlights the importance of improving biomarker detection and validation to enhance early diagnosis and personalized treatment in clinical practice.

### Future perspective

Future research on iCCA holds promise for addressing ongoing controversies and advancing treatment strategies. Disagreements persist regarding optimal surgical approaches and effectiveness of emerging systemic therapy regimens, including targeted therapies and immunotherapies, underscoring a need for robust clinical trials. Future studies should prioritize exploring the comparative outcomes of various surgical approaches, the prognostic significance of lymph node involvement, and the optimal sequence of multimodal therapies. Efforts in precision oncology, focusing on molecular profiling, could further personalize therapeutic approaches and maximize the efficacy of systemic treatments. Such research will be pivotal in refining iCCA treatment paradigms, enhancing survival rates, and improving the quality of life for patients.

## Conclusions

Hepatic resection is the only treatment able to achieve long-term survival in patients with iCCA, even though R0 resection is associated with a high rate of recurrence and consequent dismal prognosis, mainly due to advanced tumour stages at diagnosis. Referring patients with iCCA to tertiary centres has demonstrated improved outcomes with a reduction in perioperative morbidity and mortality. Multidisciplinary approach with neoadjuvant systemic or locoregional treatments, combined with increasing surgical experience and novel liver regeneration techniques, has boosted the chances of resectability of iCCA and reduced the risk of postoperative liver failure. For selected patients with exclusive liver disease, liver transplantation could be a viable option, either in early stages diagnosed in the context of chronic liver diseases or in unresectable locally advanced tumours without extrahepatic tumour spread with stable response to neoadjuvant therapy. However, future research with prospective randomized clinical trials is needed to fully understand the potential of liver transplantation for iCCA. Improved systemic therapies have also shown promising results in the adjuvant setting. Molecular profiling is gaining more attention due to the encouraging results shown by new targeted therapies in the settings of unresectable iCCA. Similarly, importance is gaining the translational research advancements providing comprehensive understanding of iCCA, integrating insights from innovative culture models, metabolomic studies, immunoprofiling, and biomarker discovery. Altogether, there is a need for continued investment in iCCA research to bridge the gap between laboratory findings and clinical practice, ultimately improving the care and survival of iCCA patients.
